# On the Diffusion of Ionic Liquids in ILs@ZIF-8 Composite Materials: A Density Functional Theory Study

**DOI:** 10.3390/molecules29081697

**Published:** 2024-04-09

**Authors:** Longlong Liu, Kun Jiang, Qingjun Chen, Lei Liu

**Affiliations:** 1 Center for Computational Chemistry, College of Chemistry and Chemical Engineering, Wuhan Textile University, Wuhan 430200, China; 2115383125@mail.wtu.edu.cn (L.L.);; 2 Key Laboratory of Rare Earths, Ganjiang Innovation Academy, Chinese Academy of Sciences, Ganzhou 341000, China

**Keywords:** ionic liquids, ZIF-8, density functional theory

## Abstract

Recently, composite materials consisting of ionic liquids (ILs) and metal–organic frameworks (MOFs) have attracted a great deal of attention due to their fantastic properties. Many theoretical studies have been performed on their special structures and gas separation applications. Yet, the mechanism for the diffusion of ILs inside MOF channels still remains unclear. Here, the DFT calculations (e.g., rigid and relaxed potential energy surface, PES, scan) together with frontier orbital analysis, natural charge analysis, and energy decomposition analysis were performed to investigate the diffusion behavior of a typical IL, [C_4_mim][PF_6_], into the ZIF-8 SOD cage. The PES profiles indicate that it is quite difficult for the cation [C_4_min]^+^ to diffuse into the cage of ZIF-8 through the pristine pores because of the large imidazole steric hindrance, which results in a large energy barrier of ca. 40 kcal·mol^−1^ at the least. Interestingly, the PES reveals that a successful diffusion could be obtained by thermal contributions, which enlarge the pore size through swing effects at higher temperatures. For example, both [C_4_mim]^+^ and [PF_6_]^−^ could easily diffuse through the channel of the ZIF-8 SOD cage when the pore size was increased to 6.9 Å. Subsequently, electronic structure analyses reveal that the main interactions between [PF_6_]^−^ or [C_4_mim]^+^ and ZIF-8 are the steric repulsion interactions. Finally, the effects of the amounts of [C_4_mim][PF_6_] on the ZIF-8 structures were investigated, and the results show that two pairs of [C_4_mim][PF_6_] per SOD cage are the most stable in terms of the interaction between energies and structural changes. With these findings, we propose that the high-temperature technique could be employed during the synthesis of IL@MOF membranes, to enrich their family members and their industrial applications.

## 1. Introduction

Recently, composite materials consisting of ionic liquids (ILs) and metal–organic frameworks (MOFs) have attracted considerable attention because of their exceptional properties [1,2]; therefore, they have been used in many important fields, e.g., gas adsorption and separation, catalysis, and sensors [3,4,5,6,7,8,9,10,11,12,13]. In particular, the large number of adsorption sites of MOFs together with ILs play an important role in the gas mixture separation (e.g., CO_2_/CH_4_) [14,15,16,17]. For example, Ban et al. [18] loaded [C_4_mim][TF_2_N] into the cages of ZIF-8, and the results showed the adsorption selectivity for CO_2_/CH_4_ significantly increased from 7.5 to 41. Following this work, Kinik et al. [19] examined the [C_4_mim][PF_6_]/ZIF-8 system via the density functional theory (DFT) and Monte Carlo (MC) simulations. The authors found that the interactions between [C_4_mim][PF_6_] and ZIF-8 created certain new adsorption sites, which increases the ideal selectivity for CO_2_/N_2_ from 7.82 to 24.21. Burak et al. [20] obtained a similar result by incorporating [C_4_mim][BF_4_] into ZIF-8, of which the selectivity of 13.3 has been obtained for CO_2_/N_2_. Afterwards, a large number of systems have been reported with different combinations of ILs and MOFs [21].

In general, the reasons for ILs@MOFs composite materials with high gas separation or selectivity have been attributed to two main aspects. On the one hand, the rich hydrogen bond network of ILs [22,23] could effectively dissolve CO_2_ molecules against other gases, e.g., CH_4_ or N_2_ [24]; hence, this improves the separation performance compared to pure MOF materials [25,26,27]. For example, Thomas et al. [28] performed DFT and Giant Canonical Monte Carlo (GCMC) simulations to investigate the selectivity of a series of IL@ZIF-8 systems. Their findings revealed that the hydrophobic fluorinated anions (e.g., [BF_4_]^−^, [Tf_2_N]^−^, and [PF_6_]^−^) exhibited higher selectivity compared to hydrophilic non-fluorinated anions (e.g., [NO_2_]^−^, [NO_3_]^−^, and [SCN]^−^). Kavak et al. [29] investigated the CO_2_ separation efficiency of five different IL-encapsulated MIL-53 systems, and they found that the CO_2_/CH_4_ separation selectivity of [C_4_mim][PF_6_]/MIL-53 was 2.8 times higher compared to the pure MIL-53. Zhang et al. [30] conducted an investigation on ILs with three different cation functional groups, and the authors revealed that ILs containing amino functional groups exhibited the highest CO_2_ molecule affinity and CO_2_/CH_4_ adsorption selectivity.

On the other hand, it is believed that the introduction of ILs could also modify the structures of the MOFs, and subsequently increase the gas selectivity. Based on the experimental techniques (e.g., high-pressure X-ray diffraction, XRD) with molecular simulations, Fairen et al. [31] demonstrated that the imidazolate linker of the ZIF-8 changed because of the swing effects, and subsequently changed the gas selectivity [32]. Uzun and colleagues [33] coated 1-(2-hydroxyethyl)-3-methylimidazolium dicyanamide ([Hemim][DCA]) on the ZIF-8 surface to eliminate the nonselective voids, and the authors found that the CO_2_ selectivity was increased by 45 times compared to the pure ZIF-8. Chang et al. [34] successfully constructed a zwitterionic MOF material (MOF UiO-66-SO_3_^−^-NH_3_^+^), and the adsorption capacity for CO_2_ increased by 32–48%, because the positive and negative charges on the modified MOF material interacted with the ILs of [Emim][SCN]. Our previous theoretical studies [35,36] revealed that the ZIF-8 aperture configurations have a significant impact on the separation of CO_2_/CH_4_. Specifically speaking, the DFT calculations showed that the pristine ZIF-8 aperture (with a pore size of 3.4 Å) exhibits the best separation performance, which has the largest energy barrier difference between CO_2_ and CH_4_; additionally, the MD simulations revealed that ILs (e.g., [C_4_mim][PF_6_]) could effectively maintain pristine aperture configurations, and retained the high separation properties.

Yet, there are still open questions following the reported mechanistic studies; that is to say, how do the ILs diffuse into the cage of MOFs (e.g., ZIF-8)? And, how many ILs could be accommodated per cage? In this vein, we performed DFT calculations to investigate the diffusion behavior of ILs into MOF cages by taking [C_4_mim][PF_6_] and ZIF-8 as an example. Firstly, the potential energy surface (PES) scan was performed to study the passing of [C_4_mim]^+^ and [PF_6_]^−^ through different ZIF-8 apertures. Secondly, several electronic analysis methods, including frontier orbital energies, natural charge analysis, and energy decomposition analysis, were used to investigate the interactions between [C_4_mim]^+^/[PF_6_]^−^ and ZIF-8. Lastly, a series of IL-encapsulated ZIF-8 with different loading amounts were investigated to obtain stabilities of ionic pairs in the SOD cage of ZIF-8.

## 2. Results

**Figure 1:**
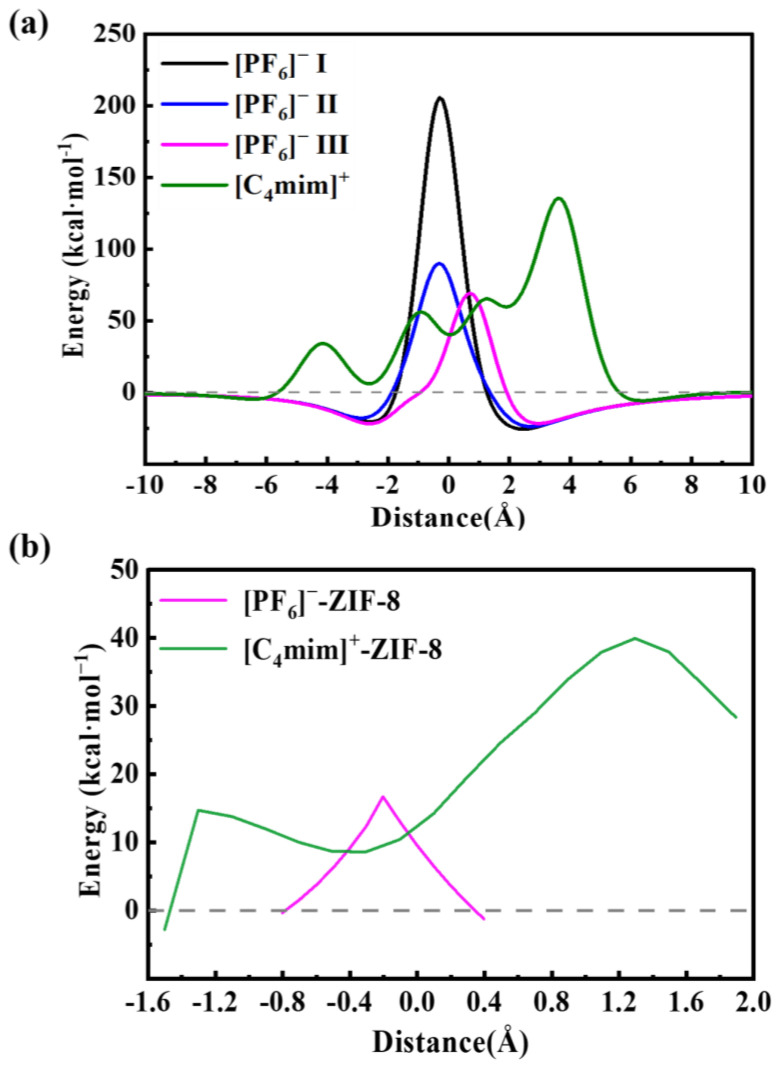
The potential energy surface curves for the anion [PF_6_]^−^ and cation [C_4_mim]^+^ passing through ZIF-8 structures: (**a**) the rigid PES scan and (**b**) the relaxed PES scan.

First of all, we performed the DFT-based PES scan calculations. In general, small energy barriers would indicate a smooth diffusion of ILs inside the pore structures of MOFs. The PES scan of the [C_4_mim]^+^ and [PF_6_]^−^ passing through the pristine ZIF-8 aperture (with a pore size of 3.4 Å) is shown in Figure 1a. It is shown that both [C_4_mim]^+^ and [PF_6_]^−^ almost cannot pass through the pore because of their large energy barriers. For [C_4_mim]^+^, an energy barrier of 135. 5 kcal·mol^−1^ was obtained, and the corresponding structure is the one when the COM of imidazole almost perpendicularly overlaps with the COM of the pristine ZIF-8 aperture. For [PF_6_]^−^, three cases have been considered (see Materials and Methods (Section 3)). The lowest energy barrier, which is 69.17 kcal·mol^−1^, was found for case **III**, in which three F atoms pointed to the plane of the ZIF-8 apertures. This finding could be explained by their molecular diameters, in which case **I** has a diameter of 3.29 Å, case **II** has a diameter of 3.20 Å, and case **III** has a diameter of 2.94 Å (Figure S1). Meanwhile, as shown in Figure S2, the results of the energy decomposition analysis show that the repulsive interactions between the [PF_6_]^−^ and ZIF-8 are the smallest when [PF_6_]^−^ adopted case **III**. After obtaining the configurations with the highest energy barriers, we then refined the PES with the relaxed scan (see Figure 1b), and we found that both [C_4_mim]^+^ and [PF_6_]^−^ still have relatively high energy barriers, being 39.87 kcal·mol^−1^ and 16.61 kcal·mol^−1^, respectively, which still prohibited [C_4_mim]^+^ and [PF_6_]^−^ from diffusing into the SOD cage of ZIF-8 at finite temperatures, e.g., room temperature. In detail, only one energy barrier was found when [PF_6_]^−^ moved from the left to the right side of the pore, and the highest energy point corresponds to the structure of which the P atom is located at the plane of the pristine ZIF-8, with three F atoms located at each side (see Figure S3a). The average distance between the H atoms of ZIF-8 and the F atoms of the [PF_6_]^−^ is computed to be 2.52 Å. While for the [C_4_mim]^+^, two energy barriers were found when [C_4_mim]^+^ moved from the left to the right of the pristine ZIF-8 aperture. The first one, 14.60 kcal·mol^−1^, corresponds to the passing of the alkyl chain, in which the average distance between the alkyl chain and the ZIF-8 aperture is 2.61 Å. The second one, 39.87 kcal·mol^−1^, corresponds to the passing of the imidazole ring, in which the average distance between the imidazole ring and the ZIF-8 aperture is only 1.66 Å (Figure S3b); hence, such small distances result in large steric hindrances or strong repulsion interactions.

**Figure 2:**
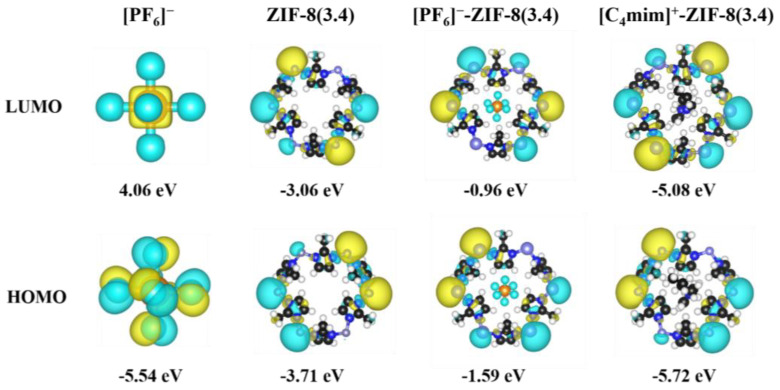
Frontier orbitals and their energies, with an isosurface value of 0.05 e/Å^3^.

Afterwards, the frontier orbitals, including the highest occupied molecular orbital (HOMO) and the lowest unoccupied molecular orbital (LUMO) were plotted in Figure 2. In general, the results indicate that there are no significant orbital interactions between the [C_4_mim]^+^/[PF_6_]^−^ and the pristine ZIF-8 aperture within the frontier orbital scheme. For example, both the LUMOs and HOMOs of [C_4_mim]^+^-ZIF-8 and [PF_6_]^−^-ZIF-8 are almost the same compared to those of the free pristine ZIF-8 aperture; in other words, the frontier orbitals mainly consist of the contributions from the ZIF-8 aperture. The HOMO–LUMO gaps of [C_4_mim]^+^-ZIF-8 and [PF_6_]^−^-ZIF-8 are also identical to those of the free pristine ZIF-8 aperture, e.g., 0.64 eV versus 0.65 eV. Moreover, the natural population analysis (NPA) was performed, and the natural charges as summarized in Table S1. The values show that a certain number of charges (ca. 0.08 *e*) have been transferred between [C_4_mim]^+^ and [PF_6_]^−^ and the pristine ZIF-8 apertures.

**Figure 3:**
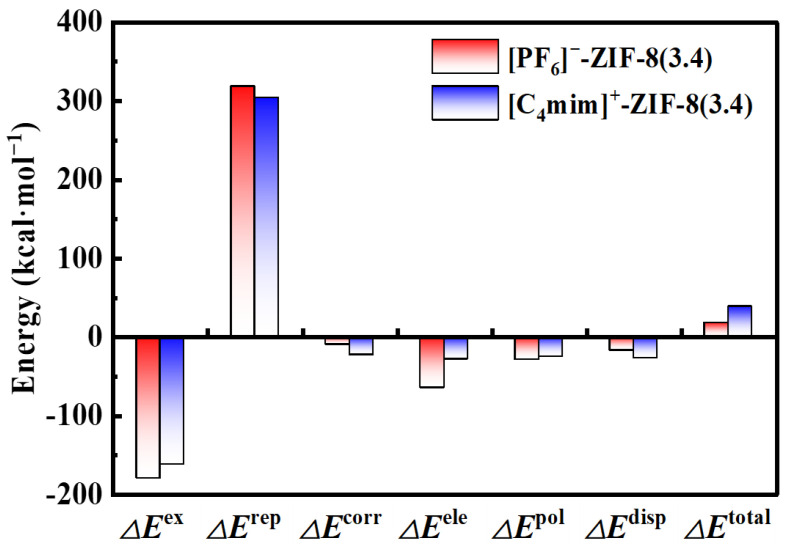
Energy decomposition analysis calculated with the GKS-EDA method at the B3LYP-D3(BJ)/6-311+G* level of theory for the [PF_6_]^−^/[C_4_mim]^+^-ZIF-8(3.4) systems.

To have a deep understanding of the interactions between [C_4_mim]^+^/[PF_6_]^−^ and the pristine ZIF-8 aperture, the GKS-EDA energy decomposition analysis has been performed based on the [PF_6_]^−^-ZIF-8(3.4 Å) and [C_4_mim]^+^-ZIF-8(3.4 Å) structures and the results are plotted in Figure 3. Generally, the interaction energies are decomposed according to the following equations:

$${\Delta E}^{total}={\Delta E}^{ex}+{\Delta E}^{rep}+{\Delta E}^{corr}+{\Delta E}^{ele}+{\Delta E}^{pol}+{\Delta E}^{disp},$$

where ${\Delta E}^{ex}$, ${\Delta E}^{rep}$, ${\Delta E}^{corr}$, ${\Delta E}^{ele}$, ${\Delta E}^{pol}$, and ${\Delta E}^{disp}$ represent exchange, repulsion, correlation, electrostatic, polarization, and dispersion components, respectively. As shown in Figure 3, the interaction energies are dominated by three terms, including the repulsion, exchange, and electrostatic energies, of which the repulsions are the main reason for the high energy barriers shown in Figure 1. The rest of the components are relatively small (e.g., less than 28 kcal·mol^−1^ in absolute values, and with a total ratio being less than 6.6%). Here, we take [C_4_mim]^+^-ZIF-8(3.4 Å) as an example and discuss those three terms individually: (1) The repulsion component. This term is responsible for steric repulsion, and it consists of the destabilizing interactions between occupied orbitals of the fragments. Usually, this term is repulsive (with a positive computed value). Here, a value of 304.27 kcal·mol^−1^ was obtained, which can be attributed to the large steric repulsion between the imidazole ring of [C_4_mim]^+^ and the pristine ZIF-8 aperture. As discussed in the PES scan section, the pristine ZIF-8 aperture has a pore size of only 3.4 Å; hence, this leaves the imidazole ring few spaces to pass through. (2) The exchange component. This term essentially is related to electrons with the same spin, exchanging their positions in degenerate orbitals to increase the stability of electronic structure states. Here, we found a value of −162.2 kcal·mol^−1^, in the case of [C_4_mim]^+^-ZIF-8. (3) The electrostatic component. This term is the energy between the unperturbed charge distributions of the prepared fragments, which is usually attractive. As shown in Figure 3, the computed electrostatic interaction is −50 kcal·mol^−1^, which is much smaller compared to the repulsion and exchange interactions. This finding is somehow consistent with the NPA charge analysis, in which the pristine ZIF-8 is slightly charged (ca. 0.08 *e*) via the charge transfer from [C_4_mim]^+^.

**Figure 4:**
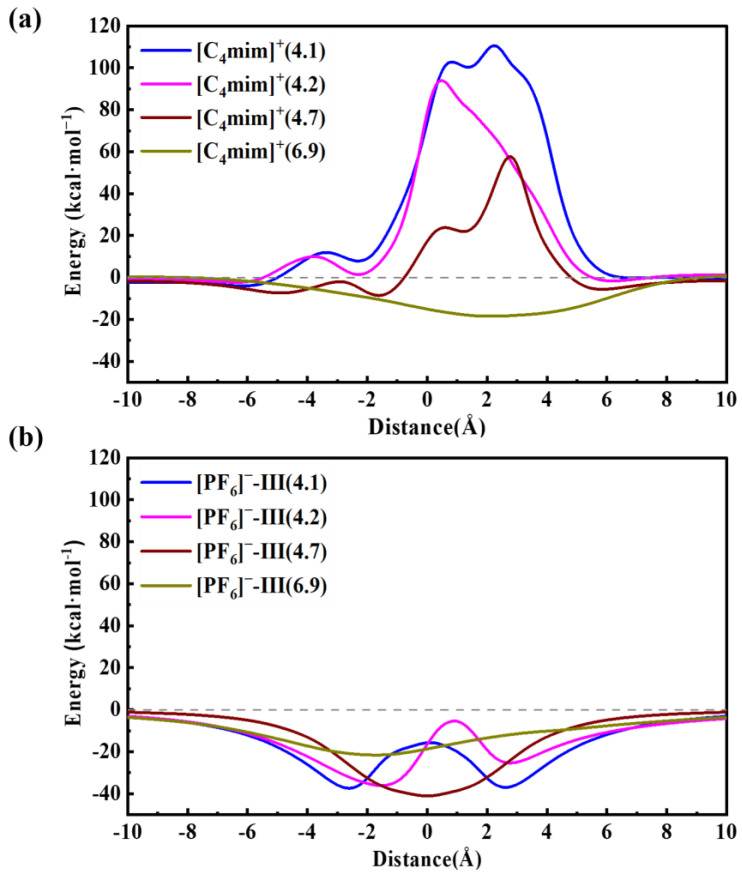
Potential energy surface curves for [C_4_mim]^+^ (**a**) and [PF_6_]^−^ (**b**) passing through different ZIF-8 apertures.

Our previous MD simulations showed that the pristine ZIF-8 aperture could be distorted because of the thermal oscillations [36]. When the simulations were performed at a temperature of 300 K, an average of 6.8 Å movements was obtained for the atoms defined by the size and shape of the pores. In other words, we could conclude that the ZIF-8 structure is very flexible against the temperatures, and the pore size might be modified by varying the synthesis temperatures. Following that study, here, we computed the PES scan of [C_4_mim]^+^ and [PF_6_]^−^ passing through the ZIF-8 aperture with the pore size of 4.1 Å, 4.2 Å, 4.7 Å, and 6.9 Å, respectively, (the pore structures are depicted in Materials and Methods (Section 3)), and the results are summarized in Figure 4. Generally, the results show that the [PF_6_]^−^ is able to freely pass through these pores with energy barriers under zero. However, [C_4_mim]^+^ can only pass through the pore with a diameter of 6.9 Å. Together with our previous room-temperature MD simulations, we conclude that a higher temperature might be needed to obtain a larger swing effect and to encapsulate [C_4_mim][PF_6_] into the ZIF-8 SOD cage. These findings are rather qualitatively consistent with a reported experiment study, in which the authors employed a two-step adsorption/infiltration method to incorporate [C_4_mim][PF_6_] into ZIF-8 [37]. In that work, [C_4_mim][PF_6_] were firstly adsorbed on the outer surface of ZIF-8, then through heat treatment at 105 °C, [C_4_mim][PF_6_] molecules infiltrated into the SOD cage. Afterwards, the temperature was cooled down to room temperature, and then the [C_4_mim][PF_6_]-encapsulated ZIF-8 composite materials were obtained.

**Figure 5:**
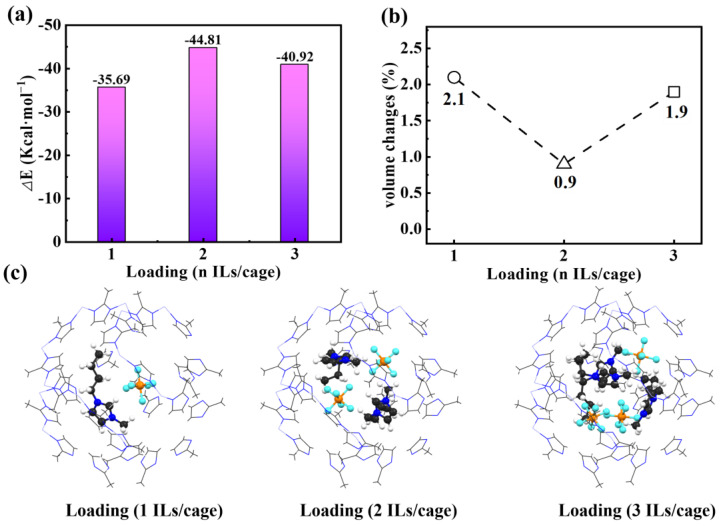
Interaction energies (**a**), volume changes (**b**), and geometric structures (**c**) of the ZIF-8 SOD cage containing one, two, and three pairs of [PF_6_][C_4_mim], respectively.

Lastly, to study how many pairs of [C_4_mim][PF_6_] could be stable inside the ZIF-8 SOD cage, and to examine the impact of their contents on the structures of the ZIF-8 apertures, the structural optimizations were performed by adding one, two, and three pairs of [PF_6_][C_4_mim] in the pristine ZIF-8 SOD cage. As shown in Figure 5a, it is shown that the interaction energies between [C_4_mim][PF_6_] and ZIF-8 were strongest when two pairs of [C_4_mim][PF_6_] were added, with a computed value of −44.81 kcal·mol^−1^, whereas the interactions energies of one and three pairs were computed to be −35.69 kcal·mol^−1^ and −40.92 kcal·mol^−1^. As such, we conclude that most of the ZIF-8 SOD cages could accommodate two pairs of [C_4_mim][PF_6_], and some of them are even able to accommodate three pairs. Overall, a value slightly larger than 2 would be obtained for the pairs of [C_4_mim][PF_6_] per SOD cage. These findings are comparable to the experimental results obtained by Ban et al. [18], in which an average of 1.4 pairs of [C_4_mim][TF_2_N] was identified per ZIF-8 SOD cage. The smaller value of 1.4 versus 2.0 could be attributed to the fact that the anion [TF_2_N]^−^ is relatively larger than [PF_6_]^−^ in space size. Compared to the experimental values [38], we found that the volume of the ZIF-8 SOD cage was increased when [C_4_mim][PF_6_] was encapsulated, of which the smallest value was obtained (0.9%) for the case with two pairs. Moreover, we also observed certain confinement effects from the results shown in Figure 5c, e.g., the structure of one pair of [C_4_mim][PF_6_] is very similar compared to the free pair (e.g., gas phase calculations), while the two and three pairs show structures closer to the condensed phase [39].

## 3. Materials and Methods

**Figure 6:**
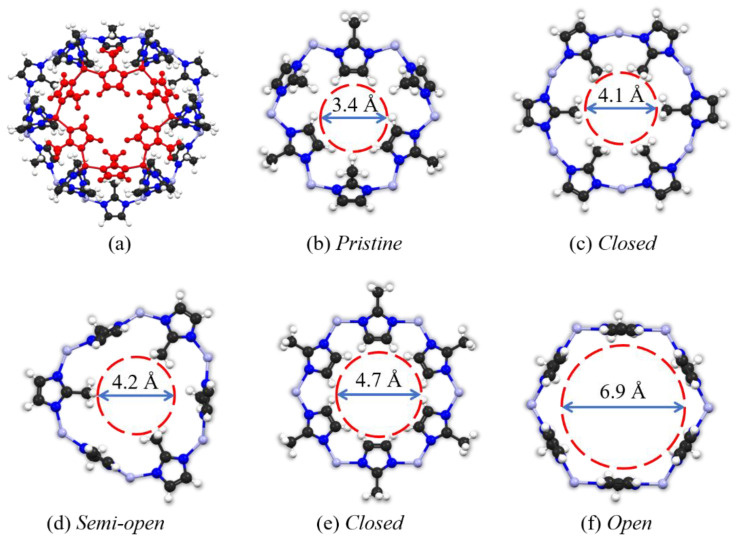
(**a**) The SOD cage presentation of the ZIF-8, and its typical (pristine) pore structure is highlighted in red color; (**b**–**f**) five different pore structures of ZIF-8 with their diameters (ref. [33]). Color legend: C, black; N, blue; Zn, light grey; H, white.

The crystal structure of the ZIF-8 was obtained from the Cambridge Crystallographic Data Centre (CCDC) Crystal Library [24], and the simplified SOD cage presentation is depicted in Figure 6a. We choose the five typical pore apertures of the ZIF-8 structure as the result of the rotation of the zinc–imidazole–zinc and methyl functional groups, as described by our previous work, [35], including “*Pristine* (3.4 Å)”, “*Closed*” (4.1 Å), “*Semi-open* (4.2 Å)”, “*Closed* (4.7 Å)”, and “*Open*” (6.9 Å), respectively, which are shown in Figure 6b–f.

**Figure 7:**
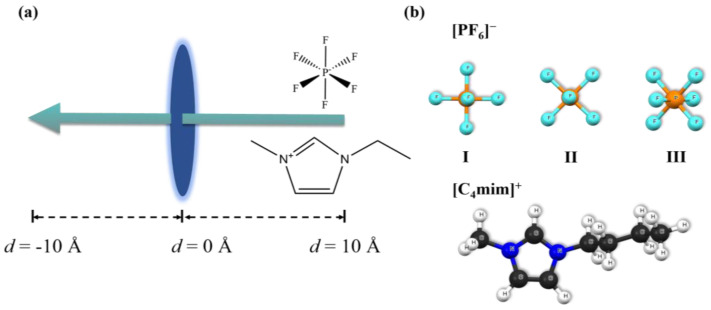
(**a**) Schematic representation of [PF_6_]^−^/[C_4_mim]^+^ passing through ZIF-8 apertures. (**b**) The geometric structures of [PF_6_]^−^/[C_4_mim]^+^, and their orientations when passing through the ZIF-8 aperture.

As demonstrated in Figure 7a, the [PF_6_]^−^ and [C_4_mim]^+^ were firstly placed 10 Å away from the aperture of the ZIF-8; then, gradually (e.g., with a step size of 0.1 Å), penetrate passed through the center of the aperture (the center of mass, COM). In principle, [PF_6_]^−^ and [C_4_mim]^+^ might have many orientations when passing through the five pore structures as shown in Figure 6. Here, we considered only the orientations that have possible low energy barriers. To summarize Figure 7b, three different orientations of [PF_6_]^−^ have been studied, denoted as case **I**, **II**, and **III**, with one, two, and three fluorine atoms (F) towards the aperture, respectively. For the [C_4_mim]^+^, one case was chosen towards the aperture, of which the imidazole ring is almost vertical to the aperture.

With the above-described models, rigid PES scans with single-point (SP) energy calculations were performed, where all atoms in the aperture of ZIF-8 and [PF_6_]^−^/[C_4_mim]^+^ were kept frozen. Afterward, we performed the relaxed PES scan within the regions near the highest energy barrier positions from the rigid PES scans (e.g., ca. −2 Å to 2 Å). In these cases, the atoms in the aperture of ZIF-8 were kept frozen, while [PF_6_]^−^/[C_4_mim]^+^ were fully optimized. Both PES scans were carried out with the Gaussian16 software package [40]. In the next discussion of both rigid and relaxed PES scans, we mainly focus on the largest energy barriers, which are the energy differences between the highest point on the PES and the free IL plus ZIF-8 structures. Subsequently, the orbital energies and charge transfer were computed with natural population analysis (NPA) [41] using the Multiwfn v.3.7 [42] software package and visualized using the VESTA v.3.5.8 [43] software package. To quantitatively study the interactions between [PF_6_]^−^/[C_4_mim]^+^ and ZIF-8, the energy decomposition analysis was performed based on the generalized Kohn–Sham energy density analysis (GKS-EDA) method [44], which is implemented in the GAMESS program package [45]. All above-mentioned DFT calculations were performed at the B3LYP level of theory, with a basis set of 6-311+G* [46], together with the damping scheme of Becke and Johnson, which denoted as DFT-D3 (BJ) [47,48].

Lastly, to study how many pairs are stable inside the SOD cage of ZIF-8, the periodic structures of ILs@ZIF-8 were examined by the BLYP functional with the dispersion correction scheme (DFT-D3). The geometry optimizations were performed to account for both changes in atomic positions and lattice dimensions, which were updated with the efficient L-BFGS algorithm. Within the DFT calculations, the DZVP-MOLOPT-SR-GTH basis set was adopted for Zn atoms, while the other atoms used the TZVP-MOLOPT -GTH basis set. The energy cutoff was set to 400 Ry, and the energy convergence for the self-consistent field (SCF) calculation was set to 1 × 10^−5^ Hartree. All those periodic calculations were performed by employing the CP2K’s Quickstep module [49].

## 4. Conclusions

In summary, the DFT calculations and several electronic structure analyses have been performed to study the diffusion behavior of the ILs, [C_4_mim][PF_6_], through the different aperture configurations of ZIF-8, and their stabilities inside the ZIF-8 cage. The results indicate that the original aperture configuration (with a 3.4 Å pore size) eventually prohibits the diffusion of [C_4_mim][PF_6_] into the SOD cage of ZIF-8, as a minimum energy barrier of 39.87 kcal·mol^−1^ had been identified, which is mainly due to steric hindrance from the imidazole ring of the [C_4_mim]^+^. The energy decomposition analysis based on the GKS-EDA method revealed that the large repulsion component is the main reason for the high energy barriers when [C_4_mim]^+^ and [PF_6_]^−^ pass through the pristine ZIF-8 aperture. Nevertheless, we found that the diffusion properties could be largely enhanced by modifying the ZIF-8 apertures, e.g., the pore size, and it is proved that this could be achieved via thermal contributions (e.g., employing a higher temperature during the synthesis procedure). Moreover, we found that two pairs of [C_4_mim][PF_6_] per SOD cage is the stable state, according to interaction energies and volume changes. Certain confinement effects were obtained as well, in which the structure of one pair of [C_4_mim][PF_6_] is similar compared to the free pair, while the two and three pairs show structures closer to the condensed phase.

## Data Availability

The original contributions presented in the study are included in the article/Supplementary Materials. Further inquiries can be directed to the corresponding authors.

## Supplementary Materials

The following supporting information can be downloaded at https://www.mdpi.com/article/10.3390/molecules29081697/s1: Figure S1: Three different ways and their diameters of the [PF_6_] pathing through the ZIF-8 pore; Figure S2: Interaction energy components calculated using the GKS-EDA method at the B3LYP-D3(BJ)/6-311+G* level for the different [PF_6_]^−^ ways towards the ZIF-8 aperture; Figure S3: The structures for the points with the highest interaction energies in the cases of [PF_6_]^−^/[C_4_mim]^+^ and ZIF-8(3.4), respectively, and the average distance refers to the closest distances between the fluorine atom on the [PF_6_]^−^, or the hydrogen atoms on the [C_4_mim]^+^ and the hydrogen atom on the aperture; and Table S1: NPA charge analysis of [PF_6_]^−^/[C_4_mim]^+^ and ZIF-8(3.4) at theoretical levels of B3LYP-D3(BJ)/6-311+G*.(values are in *e*).
