# Supplementary material for: On the Diffusion of Ionic Liquids in ILs@ZIF-8 Composite Materials: A Density Functional Theory Study
Source: Molecules. 2024 Apr 9;29(8):1697. doi: 10.3390/molecules29081697 (PMC11052405; doi:10.3390/molecules29081697)
Supplement: Supplementary file 1 [file molecules-29-01697-s001.zip › molecules-2913000-supplementary.pdf]

Supporting Information

On the diffusion of ionic liquids in ILs@ZIF-8 composite materials: a density functional theory study

Longlong Liu,[a] Kun Jiang,[a] Qingjun Chen,[b] Lei Liu[a*]

[a] Center for Computational Chemistry, College of Chemistry and Chemical Engineering, Wuhan Textile University, Wuhan, 430200 P. R. China

[b] Key Laboratory of Rare Earths, Ganjiang Innovation Academy, Chinese Academy of Sciences, Ganzhou 341000, China

Corresponding author:

Lei Liu, liulei3039@gmail.com; liulei@wtu.edu

Figure S1 Three different ways and their diameters of the [PF6] pathing through the ZIF-8 pore.

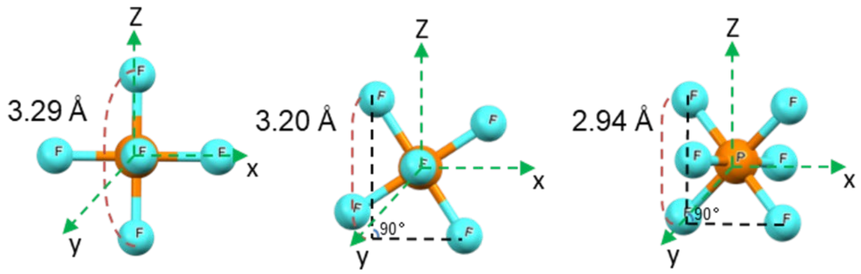

Figure S2. Interaction energy components calculated using the GKS-EDA method at the B3LYP-D3(BJ)/6-311+G* level for the different $[PF_6]^-$ ways towards the ZIF-8 aperture.

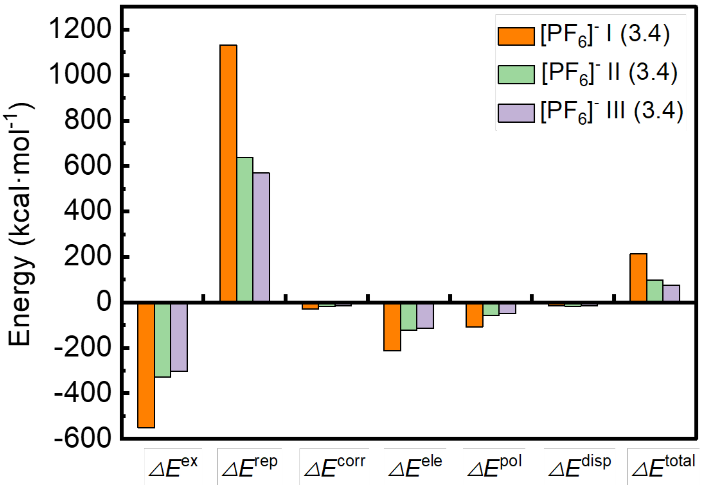

Figure S3. The structures for the points with the highest interaction energies in the cases of $[PF_6]^-/[C_4mim]^+$ and ZIF-8(3.4), respectively, and the average distance refers to the closest distances between the fluorine atom on the $[PF_6]^-$, or the hydrogen atoms on the $[C_4mim]^+$ and the hydrogen atom on the aperture.

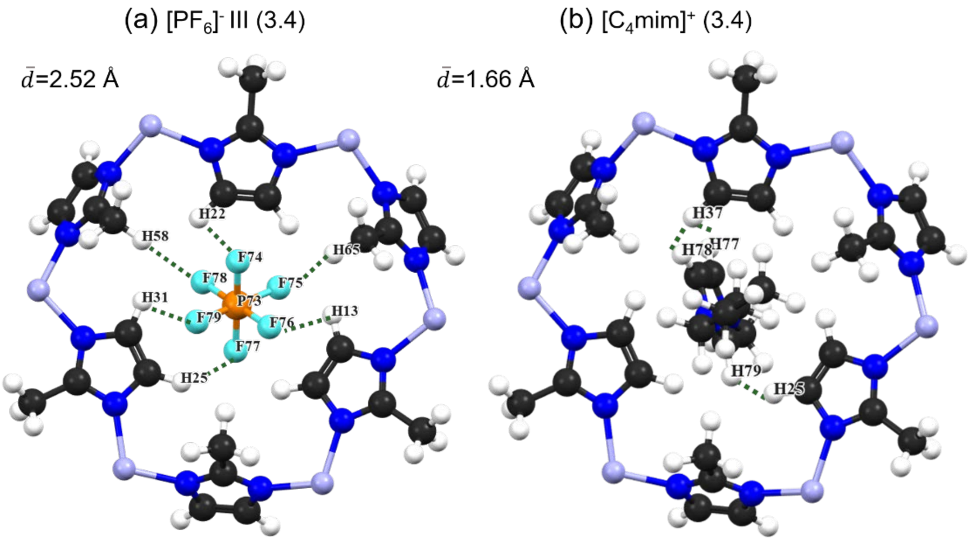

Table S1. NPA charge analysis of $[PF_6]^-$/$[C_4mim]^+$ and ZIF-8(3.4) at theoretical levels of B3LYP-D3(BJ)/6-311+G*.(values are in *e*)

System	*q*(anion)	*q*(cation)	*Δq*(ZIF-8)
**$[PF_6]^-$ III (3.4)**	**-0.914**	**--**	**-0.086[a]**
**$[C_4mim]^+$ (3.4)**	**--**	**0.916**	**0.084[b]**

*The initial charge of the ZIF-8 surface is zero.

q(anion) and q(cation) is the charge distribution of $[PF_6]^-$ and $[C_4mim]^+$ after the NPA charge calculation results, respectively.

[a]△q(ZIF-8) is the sum of the charge distribution in the $[PF_6]^-$-ZIF-8(3.4) system.

[b]△q(ZIF-8) is the sum of the charge distribution in the $[C_4mim]^+$-ZIF-8(3.4) system.
